# Preparation of a Bioadhesive Poly(Acrylic Acid)/Polyvinylpyrrolidone Complex Gel and Its Clinical Effect on Dental Hemostasis

**DOI:** 10.3390/gels8080462

**Published:** 2022-07-23

**Authors:** Tomoko Ito, Shingo Yamaguchi, Daisuke Soga, Takayuki Yoshimoto, Yoshiyuki Koyama

**Affiliations:** 1 Obara Hospital Research Institute, 3-28-16, Honcho, Nakano-ku, Tokyo 164-0012, Japan; 2 Department of Immunoregulation, Institute of Medical Science, Tokyo Medical University, 6-1-1, Shinjuku, Shinjuku-ku, Tokyo 160-8402, Japan; yoshimot@tokyo-med.ac.jp; 3 Japan Anti-tuberculosis Association, Shin-Yamanote Hospital, 3-6-1, Suwa-cho, Higashimurayama, Tokyo 189-0021, Japan; yamaguchi4168@yahoo.co.jp (S.Y.); soga_0527@yahoo.co.jp (D.S.)

**Keywords:** bioadhesion, hemostasis, poly(acrylic acid), polyvinylpyrrolidone, hyaluronic acid, hydrogel

## Abstract

Poly(acrylic acid) (PAA) is a water-soluble synthetic polymer that exhibits bioadhesive properties and has been applied in various novel medical devices, such as drug-delivery carriers and hemostatic agents. PAA forms a water-insoluble complex when mixed with polyvinylpyrrolidone (PVP). If PAA and PVP are mixed in water, they form an aggregated precipitate, which neither swells nor adheres to tissues. The formation of the hydrophobic complex was caused by hydrophobic interactions between the main chains of both polymers aligned the same as a zipper. To hinder the zipper-like alignment of the polymer main chains, hyaluronic acid (HA), a macromolecular viscous polysaccharide, was added to the PVP solution prior to complex formation. When the initial concentration of PAA was lower than 0.05%, HA effectively prevented the aggregation of PAA/PVP complexes and resulted in a slightly clouded suspension. Freeze-drying of the mixture yielded a soft white sponge, which could immediately swell in water to form a highly bioadhesive hydrogel. The PAA/PVP complex prepared with HA exhibited high hemostatic efficiency in clinical studies, even in patients on antithrombotic drugs.

## 1. Introduction

Poly(acrylic acid) (PAA) is a water-soluble synthetic polymer that is well-established as a mucoadhesive material [1]. This property of PAA enables the delivery of bioactive agents with a prolonged residence time at a specific site, and several types of PAA derivatives and complexes have been explored to develop gastrointestinal [2], buccal [3], nasal [4], vaginal [5], or ophthalmic [6] controlled drug delivery systems. PAA exhibits good adhesion properties not only to the mucosa but also to the skin [7], bone [8], and teeth [9]. In addition to drug delivery systems, the adhesive properties of PAA to biotissues have enabled the development of various novel medical devices (e.g., hemostatic agents and wound dressings) [10,11,12,13]. Recently, the need for an efficient hemostatic material has grown as increasing number of patients taking anticoagulants or antiplatelets drugs. In dentistry, hemorrhage control after dental extraction in patients receiving antithrombotic drugs is sometimes difficult. The bioadhesive dressing is expected to cover the bleeding site, and physically arrest hemorrhage without depending on the blood coagulation mechanisms.

For application to the hemostatic devices, PAA is preferably water-insolubilized. To prepare an insoluble PAA derivative, copolymerization of acrylic acid monomers with multifunctional monomers (e.g., 2,5-dimethyl-1,5-hexadiene [14] or divinyl molecules [15]) was performed to obtain three-dimensional crosslinked PAA gels. Water-insolubilization could also be attained by densely crosslinking of pre-synthesized linear PAA by γ-irradiation [16]. Those crosslinked PAAs absorb water to form swollen hydrogels. However, it is difficult to produce covalently crosslinked three-dimensional gels in a predetermined shape by injection or pressure molding. The low biodegradability of chemically crosslinked PAAs may also become a problem when they are introduced in a living body. The authors developed a water-absorbing PAA complex with polyvinylpyrrolidone (PVP) [17]. Bioadhesive hydrogels were immediately formed in water, and the resulting hydrogel slowly dissociated and re-dissolved in a neutral buffer [18]. PAA has long been known to form a water-insoluble complex upon mixing with PVP [19,20,21]. However, simple mixing of aqueous PAA and PVP solutions resulted in a non-adhesive precipitate, which was neither soluble nor swollen in water. The formation of the hydrophobic complex is caused by hydrophobic interactions between the main chains of both polymers aligned the same as a zipper [22,23]. We expected that a water-absorbing complex could be obtained if the parallel polymer main chains were disturbed. We then attempted to mix the polymers at the solid–solution interface as follows. First, a PAA film was prepared by drying an aqueous or an ethanol solution of the polymer on a plate. An aqueous PVP solution was then added to the PAA film. PAA gradually swelled into the PVP solution to form a soft swollen hydrogel, which formed a transparent PAA/PVP complex film upon drying. The dried PAA/PVP complex film swelled in water to form a bioadhesive hydrogel. The swelling PAA chains at the solid–solution interface may limit mobility and avoid the zipper-like alignment of the polymer chains, allowing the formation of a partially bonded PAA/PVP complex [17,18].

The zipper-like alignment of the polymer main chains may also be hindered by the co-presence of another water-soluble polymer during complex formation. Hyaluronic acid (HA) is a highly biocompatible macromolecular viscous polysaccharide. We attempted to prepare a water-absorbing PAA/PVP complex via the addition of HA prior to the complex formation. Freeze-drying of the mixture yielded a soft white sponge, which could immediately swell again in water to form a bioadhesive hydrogel. In this study, PAA/PVP/HA sponges were prepared under various conditions, and their swelling behavior and application as a hemostatic device for tooth extraction were examined.

## 2. Results

### 2.1. Viscosities and Turbidity of PAA- and PVP-Solutions and Their Mixtures

**Figure 1:**
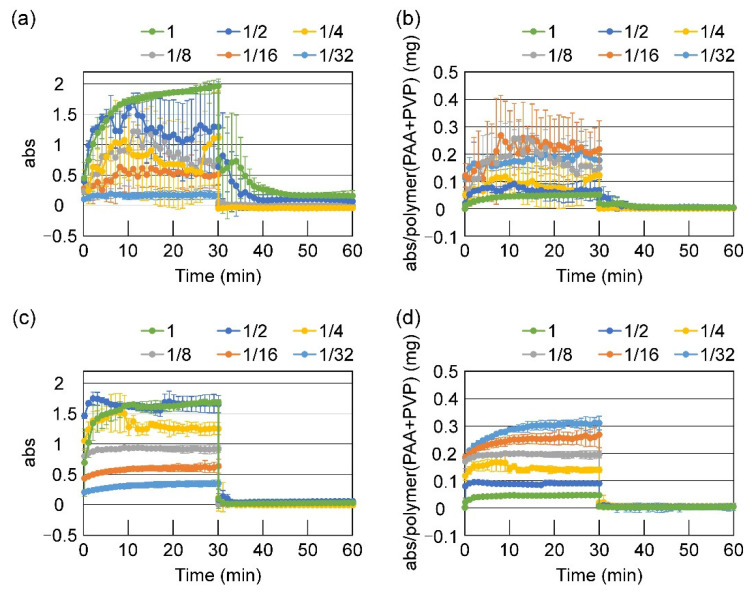
Turbidity of the PAA solutions after mixing with PVP solutions: (**a**,**b**) lightly crosslinked PAA (LC-PAA) and PVP, and (**c**,**d**) highly crosslinked PAA (HC-PAA) and PVP. Polymers were mixed in distilled water, and 30 min later, the same volume of phosphate buffer (500 mM, pH 7.4) was added.

A lightly crosslinked PAA (LC-PAA), and a highly crosslinked PAA (HC-PAA) were used in the study due to their higher biosafety than linear PAAs. They are not too densely crosslinked, and can be highly swelled in water almost to be a homogeneous solution. Serially diluted aqueous solutions of PAA (0.047–1.5%) and PVP (0.072–2.31%) were prepared, and their viscosities were measured by a type-B viscometer. As shown in Table 1, HC-PAA demonstrated lower viscosity compared to that of LC-PAA, owing to the limited spreading of the molecular chains by high density crosslinking. The pH value of LC-PAA was 2.74 at 1.5%, which was increased as the dilution rate to 4.06 at 0.047%. HC-PAA showed almost the same values (pH 2.81 at 1.5% and pH 4.44 at 0.047%). An equal volume of PVP solution was then added to the solution of LC-PAA or HC-PAA, and the turbidity of the mixture was recorded. The mixing ratio of PAA to PVP was 1:1 in moles of repeating units (1:1.54 in weight). First, PAA and PVP were mixed in phosphate buffer at pH 7.4. No clouding or precipitation was observed in any case, and the absorbance maintained similar values as the original PAA solutions (data not shown). When the polymers were mixed in distilled water, the mixed solutions became cloudy, and sometimes a white precipitate was formed. In the case of LC-PAA, it took several minutes for the mixture to become homogeneous, owing to its high viscosity, and the turbidity gradually increased (Figure 1a). HC-PAA solution was a colloidal and low-viscosity liquid, and readily mixable with the PVP solution. It immediately became cloudy upon addition of the PVP solution (Figure 1c).

Figure 1b,d demonstrate the absorbance of the mixtures per mg of the total weight of PAA and PVP. The turbidity depended significantly on the concentration in both cases with LC-PAA and HC-PAA, and mixing at the lower concentration resulted in a higher absorbance per polymer weight. The relationship is unclear in the case of LC-PAA (and is likely a result of complex precipitation). For HC-PAA/PVP mixtures, the absorbance per milligram increased with dilution. When phosphate buffer (pH 7.4) was added to the cloudy PAA/PVP mixture 30 min after mixing, the turbidity immediately decreased to almost zero in all cases, indicating that the preformed PAA/PVP complex dissociated and dissolved at pH 7.4.

**Figure 2:**
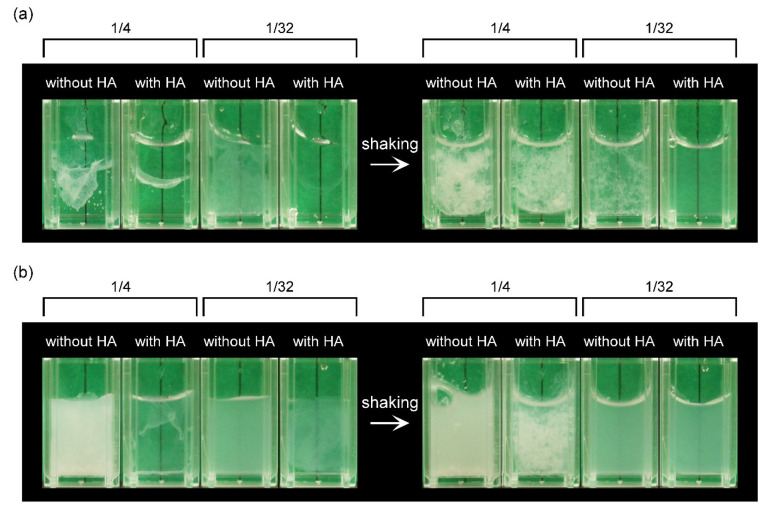
Images of the PAA/PVP solution mixtures soon after mixing in distilled water with or without hyaluronic acid (HA). PAA:PVP:HA = 1:1.54:0.8 in weight. (**a**) LC-PAA/PVP with or without HA, (**b**) HC-PAA/PVP with or without HA.

**Figure 3:**
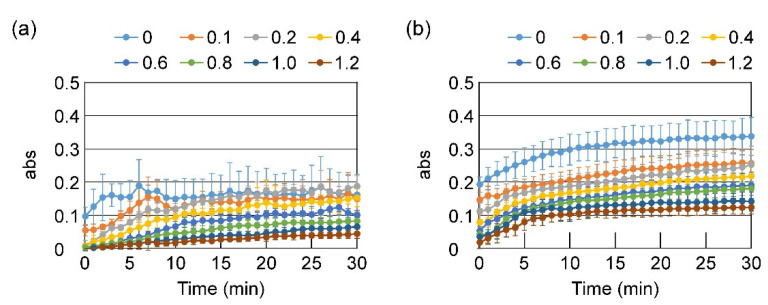
Turbidity of the PAA solutions after the addition of PVP/HA premixed solutions: (**a**) LC-PAA and PVP/HA, (**b**) HC-PAA and PVP/HA. The final concentration of PAA was 0.023%. Mixing ratio: PAA:PVP:HA = 1:1.54:0, 0.1, 0.2, 0.4, 0.6, 0.8, 1, or 1.2 in weight.

To inhibit zipper formation between the main polymer chains, causing strong hydrophobic interactions, HA with a high molecular weight was premixed with PVP prior to the addition to PAA. As shown in Figure 2, PAA and PVP formed precipitates at high concentrations also in the presence of HA. However, under diluted conditions, the pre-addition of HA suppressed clouding or precipitate formation. The influence of the amount of HA on the turbidity of the PAA/PVP mixture was examined. PVP and HA were premixed in water at various ratios and added to the 0.047% PAA solution at a final PAA:PVP:HA weight ratio of 1:1.54:0–1.2. Figure 3 illustrates the change in the absorbance of the PAA solutions after the addition of the premixed PVP/HA solutions. For both LC-PAA and HC-PAA, HA pre-addition effectively suppressed the turbidity increase, and the higher amount of HA caused the final mixtures to have lower turbidities.

### 2.2. Adhesion Strength of the PAA/PVP Sponge

**Figure 4:**
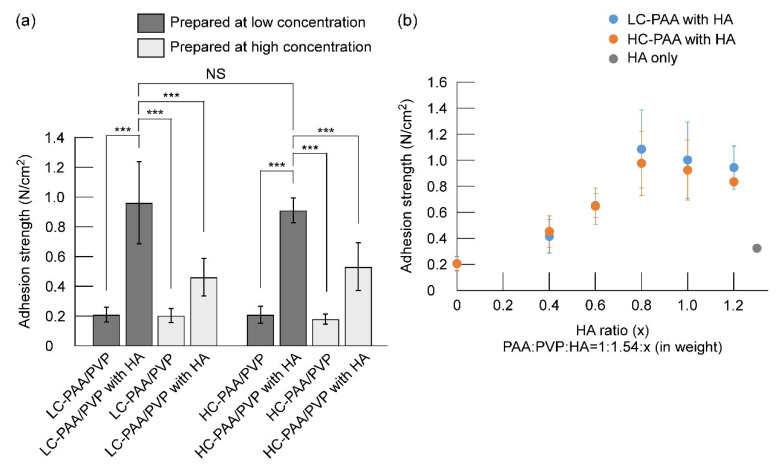
Adhesion strengths of the PAA/PVP sponges to the synthetic leather. (**a**) PAA/PVP sponges prepared with or without HA at low or high concentration: Concentrations of PAA, PVP, and HA before mixing were 0.05, 0.077, and 0.04 wt%, respectively, or 10 times higher. Significance in difference between two groups were tested by Student *t*-test. (*n* = 5, mean ± SD, *** *p* < 0.001, NS; not significant). (**b**) PAA/PVP sponges were prepared at a ratio of PAA:PVP:HA = 1:1.54:0.4–1.2. The total weight of the polymers was fixed at 2.54 mg.

PAA and PVP were mixed at low or high concentrations with or without HA (low concentration: [PAA] = 0.05% and [PVP] = 0.077%; high concentration: [PAA] = 0.5% and [PVP] = 0.77%). The mixtures were freeze-dried to afford white sponges, and their adhesion strength to the synthetic leather was measured. The difference between sponges made of HC-PAA and LC-PAA is unclear. The sponges prepared in the presence of HA exhibited a higher adhesive strength than those prepared under similar conditions without HA. The concentration of polymers in the complex formation significantly affected the adhesion strength. The complexes prepared at lower concentrations exhibited higher adhesion strengths, and the PAA/PVP complex prepared at a low concentration in the presence of HA exhibited the highest adhesion strength of approximately 1 N/cm^2^ (Figure 4a).

The influence of the HA/PAA ratio of the complex preparation solution on the adhesive strength was measured using sponges of the same weight (i.e., 2.54 mg). The adhesive strengths of the PAA/PVP sponge prepared without HA was approximately 0.2 N/cm^2^, and that of HA alone was 0.3 N/cm^2^. The addition of HA significantly improved the adhesive strength of the PAA/PVP complex depending on the HA content, and the highest strength was obtained at an HA/PAA ratio of 0.8 (Figure 4b).

### 2.3. Evaluation of the Cytotoxicity of PAA/PVP Complexes

**Figure 5:**
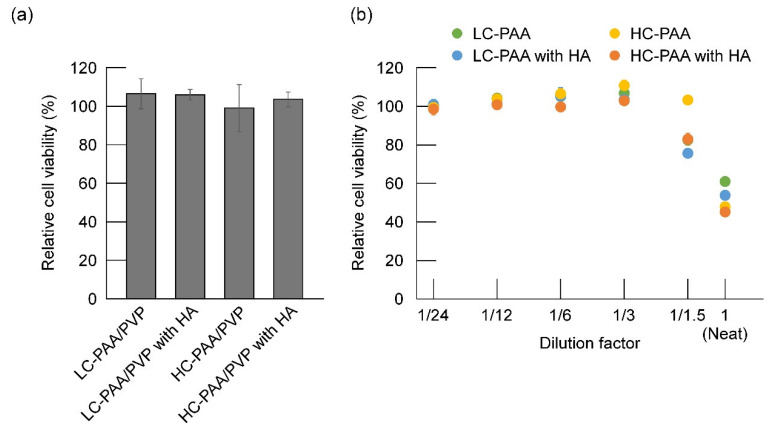
Viability of fibroblasts treated with (**a**) solid PAA/PVP sponges and (**b**) extract solutions from the PAA/PVP complexes. Data were presented as a relative cell viability compared with control cells to which just medium was added instead of the sponge or extract.

PAA/PVP sponges with or without HA were placed directly on the cultured fibroblasts, and their cytotoxicity was examined. The sponges were placed on the cells, and the medium was gently added to the wells. The sponges were immediately swollen in the wells. After overnight incubation, the cells were observed under a microscope. Living cells attached to the gels were observed at their edges. The cells were also observed in the swollen gels. The WST-1 assay was performed, and the data were presented in Figure 5a as a relative cell viability compared with control cells to which just medium was added instead of the sponge. As shown in the figure, the swollen gels caused no decrease in the cell number, regardless of the type of PAA or the presence or absence of HA.

The extract of the PAA/PVP complexes was prepared by immersing the complex overnight in 1 mL of cell culture medium, and the cytotoxicity of the extract on fibroblasts was also evaluated. Figure 5b presented the relative cell viability compared to control untreated cells after 20 h of incubation with dilution factors. Irrespective of the crosslinking level of the PAAs or whether HA was included, cytotoxicity of the extracts was very low, and 100% survival of the cells was observed in the three times or more diluted extracts.

### 2.4. Re-swelling Behavior of the PAA/PVP Complex Sponge in Water

**Figure 6:**
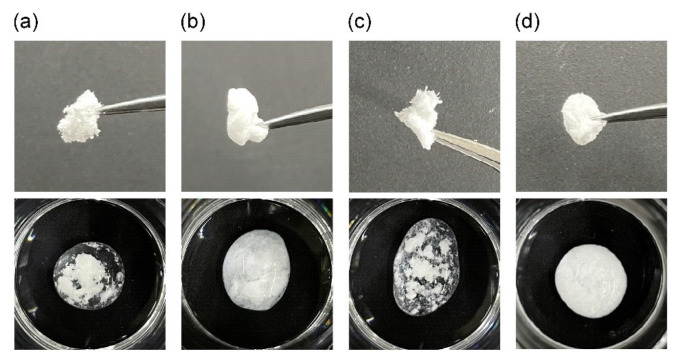
Images of the freeze-dried PAA/PVP complexes (upper), and 30 min after the addition of water (lower). (**a**) LC-PAA/PVP complex prepared without HA, (**b**) with HA, and (**c**) HC-PAA/PVP complex prepared without HA, and (**d**) with HA.

The sponges prepared without HA were weak and brittle, whereas those containing HA were smooth and flexible. After immersion in water, the sponges without HA instantly aggregated and precipitated (Figure 6a,c). The LC-PAA/PVP sponge prepared with HA swelled slightly (Figure 6b). Meanwhile, the HC-PAA/PVP complex sponge prepared in the presence of HA rapidly absorbed water to become a swollen hydrogel and spread out in a water drop (Figure 6d).

### 2.5. Hemostatic Effect of the PAA/PVP Sponge on the Incised Femoral Vein of a Mouse

**Figure 7:**
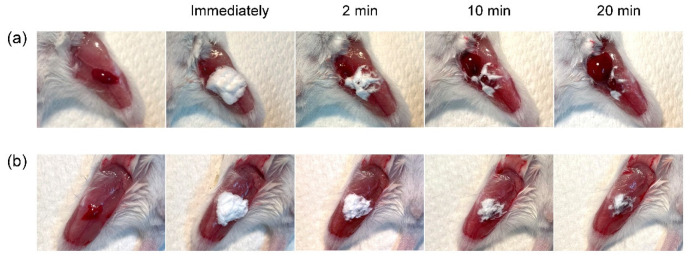
Hemostatic effect of the PAA/PVP complex sponges prepared in the presence of HA on femoral vein of mouse injected with low-molecular-weight heparin (fragmin; 20IU): (**a**) LC-PAA/PVP with HA and (**b**) HC-PAA/PVP with HA.

The PAA/PVP sponges prepared without HA were brittle and difficult to handle, and their adhesion strength was low, as mentioned previously. The LC-PAA/PVP or HC-PAA/PVP sponges prepared with HA were used in subsequent hemostatic experiments. The femoral vein of each mouse was exposed and cut, and 3.34 mg of LC-PAA/PVP/HA or HC-PAA/PVP/HA sponge was placed on the bleeding site. The LC-PAA/PVP/HA sponge absorbed blood and aggregated within minutes. Blood leakage was observed through the precipitated complex, and hemostasis was not obtained. However, the HC-PAA/PVP/HA sponge formed an adhesive hydrogel and bonded tightly to the hemorrhage site. Complete hemostasis was achieved immediately, and no bleeding was observed for >20 min (Figure 7).

### 2.6. Clinical Study on the Hemostasis after Tooth Extraction

**Figure 8:**
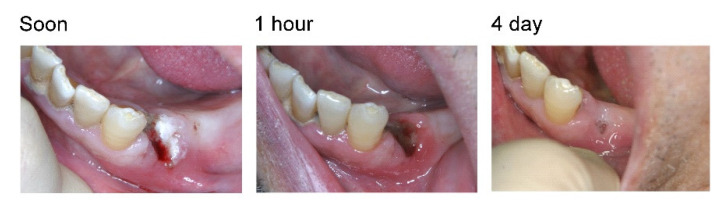
Images of the tooth sockets after extraction, followed by treatment with HC-PAA/PVP/HA sponge (Patient No 6).

The HC-PAA/PVP complex sponges were prepared in the presence of HA and used in clinical studies to evaluate the hemostatic effect after tooth extraction. The studies were performed in six patients, five of whom were taking antithrombotic drugs (Table 2). HC-PAA/PVP sponge was inserted in the bleeding socket after tooth extraction. In all cases, complete hemostasis was achieved immediately after insertion of the sponge in the tooth socket, and post-bleeding was not observed in any patient. The sockets treated with the HC-PAA/PVP complex healed after several days (Figure 8).

## 3. Discussion

When PAA and PVP were mixed in water, an insoluble polymer complex formed and precipitated. Drying the complex yielded a white solid that was neither dissolved nor swollen in water. The solid complex does not adhere to bio-tissues and thus cannot be applied to bio-adhesive medical devices. The PAA/PVP complex has been known as a hydrogen-bonding polymer complex. However, thermodynamic analysis of their complex formation behavior revealed that the entropy change was positive for the complexing process. This suggests that hydrophobic interaction between the polymers is another one of the main driving forces for the formation of water-insoluble complexes [23]. Hydrophobic bonding is a relatively weak intermolecular force, and large hydrophobic groups are required to provide a strong binding force. Among the PAA and PVP structures, only the main chains of both polymers consisting of carbon and hydrogen atoms are large hydrophobic groups. To produce a strong hydrophobic bonding force, the main chains of the polymers should be aligned to form a zipper structure. A zipper structure in the hydrogen-bonding polymer complex has also been proposed to explain the thermodynamic stability of the polymer complexes [24] In the process of complex formation between PAA and PVP, after the first contact and random binding, segments of both polymers would slide or exchange with another segment to solve loops or misfits. When the aqueous polymer solutions are mixed, both polymer chains can move easily in water, and a firm hydrophobic-bonded complex with a zipper structure is formed. Polymer-polymer complexes have a tendency, in general, to stick to one another to a large aggregate. Molecular weight and concentration much affect the aggregation behavior [25,26], but it is difficult to completely prevent the phase separation only by dilution and molecular weight decrease. As shown in Figure 1, PAA/PVP complex aggregated and clouded the mixture even at low concentrations.

A swellable complex gel would be obtained by hindering the free movement of the polymer chains to inhibit the rearrangement of the bound segments. Bekturov et al. prepared a highly hydrated PAA/PVP complex using the chemically crosslinked PVP gel [27]. They made a three-dimensional cross-linked PVP gel by the reaction of linear PVP with epichlorohydrin. The crosslinked PVP gel was swollen in water, and then immersed in the aqueous solution of PAA. PAA penetrated into the gel, and formed hydrogen bonding with PVP chains. However, the swollen PVP gel did not shrink, but remained as a highly swollen state, owing to the limited movement of the PAA chains inside the network structure. The PVP-gel/PAA complex exhibited characteristic swelling behavior responsive to pH, and it may be utilized for stimuli-induced drug delivery systems. But, the chemical cross-linkages are not degraded in living body, and thus, such a PAA/PVP complex hydrogel is not applicable to an implantable medical device. In previous studies, we prepared a water-swellable and biodissociable PAA/PVP complex through the solid–solution interface complex formation by adding PVP solution to the dried PAA film formed on the bottom of the vessel [17,18]. The PAA chains swelled into the PVP solution. However, the movement of the PAA chains was strictly limited by the immobilized segments in the solid state. Subsequently, a highly swollen PAA/PVP complex gel was obtained, which was re-swellable in water after drying. The re-swollen hydrogel exhibited high bioadhesive properties and favorable hemostatic effects in mice [17,18]. When the dried PAA/PVP complex film was placed in the peritoneal cavity of mice, it immediately formed a swollen gel, and was slowly dissociated and dissolved out in a biological fluid in a week.

Steric hindrance by the presence of a third water-soluble polymer may be another method to hamper the free movement of the PAA and PVP polymer chains. In this study, we examined the effect of the coexistence of high molecular weight HA at the time of complex formation. First, aqueous solutions of PAA and PVP were mixed at various concentrations in the absence of HA. When LC-PAA was mixed with PVP, the mixture became cloudy within 5 min and formed a precipitate, regardless of the concentration (Figure 1a,b). Dilution of the solutions did not prevent precipitation. The relationship between the concentration and absorbance per weight of polymer is obscure, likely being caused by the partial precipitation of the complex. As shown in Table 1, the solution of HC-PAA is a low-viscosity liquid owing to the small excluded volume of the highly crosslinked polymer and became cloudy as soon as the PVP solution was added (Figure 1c,d). Mixing of the solutions of higher concentrations resulted in lower absorbance per weight, likely owing to the heterogeneity of the mixture, which was caused by the aggregation and partial precipitation of the polymer complexes.

The effect of pre-addition of HA to the PAA solution was then examined. As shown in Figure 2, at higher concentrations, the coexistence of HA could not prevent the aggregation and precipitation of the complex for both LC-PAA and HC-PAA. However, when the initial concentrations of PAAs (before mixing) were lower than 0.05%, HA effectively prevented the aggregation and precipitation of the PAA/PVP complexes (Figure 2). In the case of LC-PAA, a lightly clouded homogeneous mixture was obtained at HA/PAA = 0.8 in weight (Figure 2a). The amount of HA significantly affected the turbidity of the mixture, and a higher HA/PAA ratio led to a lower turbidity for both PAAs (Figure 3).

PAA is known as a bioadhesive water-soluble polymer. However, the sponges prepared by simple mixing of the aqueous solutions of PAA and PVP followed by freeze-drying did not exhibit bioadhesive properties, regardless of the crosslinking density of PAA and the mixing concentration. The carboxylic acid groups in the PAA side chains play an important role in adhesion [14,28]. In the PAA/PVP complex, where both polymer chains are aligned in a zipper-like structure [22], most carboxyl groups are consumed by hydrogen bonding with the pyrrolidonyl groups of the PVP molecules [23]. In contrast, in the complex obtained by mixing PAA and PVP in the presence of HA, the parallel lining of PAA and PVP was inhibited, and not a little carboxyl groups remained in the nonbinding state. The adhesion strength of the PAA/PVP complex to the synthetic leather film was measured. As expected, the adhesion strength of the complexes formed in the presence of HA was significantly higher than those prepared without HA (Figure 4), and sponges prepared from LC-PAA and HC-PAA at low concentrations with HA exhibited high adhesive strengths (approximately 0.9 N/cm^2^). As previously mentioned, a higher amount of HA resulted in a lower turbidity of the mixture. However, the adhesion strength decreased when HA was added at HA/PAA > 0.8 in weight (Figure 4b), likely due to the fact that excessive HA content in the complex would lead to a low density of carboxyl groups. Thus, the following experiments were performed with sponges prepared at HA/PAA = 0.8, where the highest adhesive strength was obtained.

The hemostatic effect of the water-absorbing sponges prepared with HA was then examined in the incised femoral veins of mice. Both LC-PAA/PVP and HC-PAA/PVP sponges containing HA immediately absorbed the blood and adhered tightly to the tissue. However, in the site treated with the LC-PAA/PVP sponge, blood leakage was observed at the top and edge of the swollen gel in 2 min, and a hemostatic effect was not observed. In contrast, HC-PAA/PVP immediately formed a homogeneous hydrogel and covered the hemorrhaging site to effectively arrest bleeding (Figure 7). As shown in Figure 6, the LC-PAA/PVP sponge did not swell, and a heterogeneous gel was formed. A non-homogeneous porous gel may contain a passage to allow the leakage of blood. Light crosslinking would leave a long free segment of the PAA chain, which would easily move and allow the formation of a zipper-like structure and partial precipitates, even in the presence of HA.

Recently, the number of patients taking anticoagulants or antiplatelets has rapidly increased due to an increase in the elderly population. Local hemostasis after tooth extraction can be challenging, particularly in patients taking antithrombotic drugs. In some cases, patients hemorrhage again after going home, and the development of an effective local hemostatic device is required. The bioadhesive hydrogel would cover the hemorrhaging spot and prevent bleeding physically, regardless of the physiological coagulation mechanisms. Thus, it could be an effective hemostatic device for patients taking antithrombotic drugs, such as warfarin and aspirin. A clinical trial for hemostasis after tooth extraction was then performed in six patients using the HC-PAA/PVP complex sponge prepared with HA. Immediate complete hemostasis was achieved in all six cases, including patients taking antithrombotic drugs. The HC-PAA/PVP complex sponge could adhere to the socket itself and did not require ligation, which would impair the local blood circulation. The swollen complex hydrogel slowly dissolved in pH neutral saliva, and disappeared within days without need for removal.

The soft hydrogel of the low-toxicity PAA/PVP complex promoted rapid and clean healing. The PAA/PVP complex swollen in the socket by absorbing the blood would contain platelets inside, which would release growing factors and accelerate healing. An investigation of the healing effects and detailed mechanisms of the gel is currently ongoing.

## 4. Conclusions

Mixing the aqueous solutions of PAA and PVP resulted in the formation of a precipitate that was insoluble in water. In contrast, mixing the polymers in the presence of HA yielded a slightly cloudy suspension, resulting in a water-absorbing fluffy sponge after freeze-drying. The sponge formed a soft hydrogel on the wet tissues and strongly adhered to them. In clinical studies after tooth extraction, the sponge effectively arrested the hemorrhage, even in patients taking antithrombotic medication. These bio-adhesive complexes have significant potential as hemostatic agents for cases with difficult hemostasis.

## 5. Materials and Methods

### 5.1. Materials

A lightly crosslinked PAA (LC-PAA), Carbopol 941, was obtained from CBC Co., Ltd. (Tokyo, Japan). A highly crosslinked PAA (HC-PAA), Carbopol 934P NF, was purchased from Kobayashi Perfumery Co., Ltd. (Tokyo, Japan). PVP (Mw = 40,000 g/mol), Kollidon 30, was provided by BASF Japan Ltd. (Tokyo, Japan). Sodium hyaluronate (i.e., HA) fermented from Streptococcus zooepidemicus HA-LQH (Mw = 1,200,000–2,200,000 g/mol) was produced by Kewpie Corporation (Tokyo, Japan).

### 5.2. Viscosity Measurements of PAA- and PVP-Solutions

Aqueous solutions of PAA (1.5%) and PVP (2.31%) were prepared, and serially double diluted by deionized water. The viscosities of the solutions were measured by a B-type viscometer (Model BM-Viscometer; Tokyo Keiki Co. Ltd., Tokyo, Japan) at 25 °C.

### 5.3. Turbidity Measurements of PAA- and PVP-Solution Mixtures

Solutions of LC-PAA or HC-PAA (50 μL) at various concentrations in distilled water were placed in the wells of a transparent 96 well plate, and then PVP solutions (50 μL in water) were added. The mixing ratio of PAA to PVP was fixed at 1:1 in moles of repeating units (1:1.54 in weight). The turbidity of the mixture was measured at a wavelength of 560 nm using a microplate reader (XFluor4GENiosPro; Tecan, Zürich, Switzerland) and recorded after every minute with 5 s of shaking before each measurement. The concentrations of the solutions are listed in Table 1. Further, 30 min after mixing, 100 μL of phosphate buffer (500 mM, pH 7.4) was added to the mixture, and turbidity measurements were obtained for another 30 min.

The influence of HA addition on the turbidity of the PAA/PVP mixtures was also examined. PAA solution (0.047%, 50 μL water) was added to each well of a 96-well plate. The same volume of solution containing both PVP (0.072%) and HA (0.0047–0.056%) was added, and the turbidity was measured for 30 min.

### 5.4. Measurement of the Adhesion Strength of the PAA/PVP Sponge

PAA/PVP sponges with or without HA for adhesion strength measurements were prepared by mixing them in distilled water followed by freeze-drying as follows. A solution of LC-PAA or HC-PAA (0.05%, 2 mL) was mixed with HA (0.04%, 2 mL) and a then with 0.077% PVP (2 mL). The samples were thoroughly mixed and freeze-dried. Sponges prepared at higher concentrations were similarly prepared by mixing 0.2 mL of each solution at 10 times higher concentrations than initially used. For the PAA/PVP sponge without HA, the same volume of water was added, instead of the HA solution.

The adhesion strengths of the complex sponges were measured, as described in a previous study [18], except that a square piece of synthetic leather, Protein Leather TM PBZ13001-BK (IDEATEX JAPAN Co., Ltd., Tokyo, Japan) (10 × 10 × 0.5 mm^3^), was used as an adhesion substrate. The PAA/PVP sponge was sandwiched between two protein leather pieces: one was fixed to a 1 kg metal weight with glue, and the other was fixed to the plastic piece with a hook for pulling. The leather pieces were moistened with wet paper prior to use. After standing for 10 s, they were placed on an electronic balance, and the protein leather on the plastic piece was pulled upward at a speed of 0.2 cm/s. The weight indicated on the balance at the moment of peeling was recorded as the adhesion strength. The effect of the amount of HA on the adhesion strength was also investigated with a similar method using sponges made with various amounts of LC-PAA or HC-PAA (0.05%), PVP (0.07%), and HA (0.04%) (PAA:PVP:HA = 1:1.54:0.4–1.2 in weight ratio). The total weight of the polymers (PAA + PVP + HA) in the sponge used in the measurement was fixed at 2.54 mg.

### 5.5. Evaluation of the Cytotoxicity of PAA/PVP Complexes

PAA/PVP complex sponges with or without HA were prepared from solutions of PAA (0.05%), PVP (0.077%), and HA (0.04%), and the cytotoxicity of the sponges was examined using two methods: direct contact of the solid sponge and extraction evaluation. A direct contact test was performed by placing the solid sponge directly onto the cultured cells as follows. Mouse fibroblasts were generated from BALB/c mice, as previously described [18]. Cells were seeded onto 24-well plates at 7 × 104 cells per well and cultured overnight in Dulbecco’s modified Eagle’s medium (DMEM) supplemented with 10% fetal bovine serum (FBS), penicillin G sodium (100 unit/mL), and streptomycin sulfate (0.1 mg/mL). After removal of the primary growth medium, a PAA/PVP sponge containing 1 mg PAA, 1.54 mg PVP, with or without HA (0.8 mg), was placed on the cells. Fresh DMEM (2 mL) with FBS and antibiotics was gently added to the wells, and the cells were incubated at 37 °C with 5% CO_2_. After 20 h of incubation, the culture medium was removed, and cell viability was measured using the WST-1 assay.

Cytotoxicity was also assessed in the medium extracts of the complexes. The PAA/PVP complex (7.62 mg, PAA:PVP = 1:1.54 in weight) or PAA/PVP complex containing HA (10.02 mg, PAA:PVP:HA = 1:1.54:0.8 in weight) was immersed in 1 mL of DMEM containing 10% FBS, penicillin G sodium (100 unit/mL), and streptomycin sulfate (0.1 mg/mL). The mixture was stirred for 1 min using a vortex mixer and incubated overnight at 37 °C. The mixture was then stirred gently and centrifuged at 5000 rpm for 10 min. The supernatant was collected and serially diluted. Fibroblasts were seeded onto 96-well plates at 8.3 × 103 cells per well and cultured overnight in DMEM supplemented with FBS and antibiotics. The medium was removed, and the complex extract (150 μL) was added to the cells. Cytotoxicity was measured after 20 h of incubation as described above.

### 5.6. Re-Swelling Behavior of the PAA/PVP Complex Sponge in Water

PAA/PVP sponges with or without HA were prepared similarly as above from solutions of PAA (0.05%), PVP (0.077%), and HA (0.04%) (PAA:PVP:HA = 1:1.54:0 or 0.8 in weight). They were immersed in distilled water, and the re-swelling behavior was observed.

### 5.7. Hemostatic Effect of PAA/PVP Complexes on Mice

All animal experiments were conducted in accordance with the protocol approved by the Tokyo Medical University Animal Committee (no. R3-0122). Female BALB/c mice were purchased from SLC (Shizuoka, Japan). The procedure was performed under general anesthesia. A vein of the femur of mice (15 weeks old) was cut, and the PAA/PVP/HA sponge (3.34 mg) prepared from solutions of PAA (0.05%), PVP (0.077%), and HA (0.04%) (PAA:PVP:HA = 1:1.54:0.8 in weight) was placed on the hemorrhage part. A hemostatic effect was observed for 20 min.

### 5.8. Clinical Study of the Hemostatic Effect of PAA/PVP Complexes

This study was approved by the Ethics Committee of Shin-Yamanote Hospital (Protocol No: 12004). A clinical study of the hemostatic efficacy of the PAA/PVP complexes after tooth extraction was performed in six patients (one male and five females, 50–87 years of age) containing those who are taking an anticoagulant or antiplatelet drug. The details are presented in Table 2. Pharmaceutical grade sodium hyaluronate (from chicken comb) produced by Kewpie Corporation (Tokyo, Japan) was used in clinical research. Soon after tooth extraction, an HC-PAA/PVP sponge (13 mg) with HA prepared from solutions of HC-PAA (0.05%), PVP (0.077%), and HA (0.04%) (PAA:PVP:HA = 1:1.54:0.8 in weight) was inserted in the bleeding socket, and hemostatic effects and recovery conditions were observed.

## Figures and Tables

**Table 1:** Concentration of the poly(acrylic acid) (PAA) and polyvinylpyrrolidone (PVP) solutions before mixing.

Dilution Factor	PAA			PVP	
	Concentration (%)	LC-PAA	HC-PAA	Concentration (%)	Viscosity (mPa·s)
		Viscosity (mPa·s)	Viscosity (mPa·s)		
1	1.5	3400	1815	2.31	3.9
1/2	0.75	1476	36	1.16	3.5
1/4	0.38	892	8.0	0.58	3.2
1/8	0.19	502	5.4	0.29	2.0
1/16	0.094	215	4.4	0.14	2.9
1/32	0.047	84	4.0	0.072	2.9

The viscosity was measured by a B-type viscometer at 25 °C. LC-PAA: lightly crosslinked PAA; HC-PAA: highly crosslinked PAA.

**Table 2:** Patients for the clinical study.

Patient	Age	Sex	Antithrombotic Drugs Taken by the Patient	Number of Extracted Tooth
1	59	Female	-	1
2	50	Female	Warfarin (INR 1.59)	2
3	52	Female	Warfarin (INR 2.0)	1
4	86	Female	Warfarin (INR 1.69)	1
5	81	Female	Bayaspirin	2
6	87	Male	Plavix	1

## Data Availability

Not applicable.
